# Supplementary material for: Practical Barriers and Facilitators Experienced by Patients, Pharmacists and Physicians to the Implementation of Pharmacogenomic Screening in Dutch Outpatient Hospital Care—An Explorative Pilot Study
Source: J Pers Med. 2020 Dec 21;10(4):293. doi: 10.3390/jpm10040293 (PMC7767378; doi:10.3390/jpm10040293)
Supplement: Supplementary file 1 [file jpm-10-00293-s001.zip › Table S5 Alphabetical list of abbreviations.docx]

**Table S5** Alphabetical list of abbreviations

| **Abbreviation** | **Explanation** |
| --- | --- |
| ADR | Adverse drug reaction |
| CDS | Clinical Decision Support |
| DPWG | Dutch Pharmacogenetics Workinggroup |
| EHR | Electronic Health Record |
| GP | General practitioner |
| ICT | Information and communications technology |
| PGx | Pharmacogenomics |
| T1, T2 | Timepoint 1, timepoint 2 |
| UMCG | University Medical Center Groningen |
